# Comparative Evaluation of Dental Clinical Surface Treatments for Polyetheretherketone with Airborne-Particle Abrasion, Hydrofluoric Acid Etching, and Handheld Nonthermal Plasma Activation on Long-Term Bond Performance

**DOI:** 10.3390/polym17111448

**Published:** 2025-05-23

**Authors:** Szu-Yu Lai, Szu-I Lin, Chia-Wei Chang, Yi-Rou Shen, Yuichi Mine, Zih-Chan Lin, Mei-Ling Fang, Chia-Chih Sung, Chien-Fu Tseng, Tzu-Yu Peng, Chiang-Wen Lee

**Affiliations:** 1 School of Dentistry, College of Oral Medicine, Taipei Medical University, Taipei 11031, Taiwan; 2 Department of Dentistry, Taoyuan General Hospital, Ministry of Health and Welfare, Taoyuan 33004, Taiwan; 3 Division of Family Dentistry, Department of Dentistry, Taipei Medical University Hospital, Taipei 11031, Taiwan; 4 Department of Medical Systems Engineering, Hiroshima University Graduate School of Biomedical and Health Sciences, Hiroshima City 734-8553, Japan; 5 Department of Nursing, Division of Basic Medical Sciences, and Chronic Diseases and Health Promotion Research Center and Center for Drug Research and Development, Chang Gung University of Science and Technology, Chiayi 613016, Taiwan; 6 Research Center for Tooth Bank and Dental Stem Cell Technology, College of Oral Medicine, Taipei Medical University, Taipei 11031, Taiwan; 7 Center for Environmental Toxin and Emerging-Contaminant Research, Cheng Shiu University, Kaohsiung 83347, Taiwan; 8 Department of Civil Engineering and Geomatics, Cheng Shiu University, Kaohsiung 83347, Taiwan; 9 Graduate Institute of Clinical Dentistry, School of Dentistry, College of Medicine, National Taiwan University, Taipei 10048, Taiwan; 10 Department of Respiratory Care, Chang Gung University of Science and Technology, Chiayi 613016, Taiwan; 11 Department of Orthopaedic Surgery, Chang Gung Memorial Hospital, Chiayi 61363, Taiwan; 12 Department of Safety Health and Environmental Engineering, Ming Chi University of Technology, New Taipei City 24301, Taiwan

**Keywords:** polyetheretherketone, light-curing cement, shear bond strength, resin bonding, handheld nonthermal plasma treatment, hydrofluoric acid etching, artificial aging

## Abstract

Polyaryletherketone (PAEK) materials, including polyetheretherketone (PEEK) and polyetherketoneketone (PEKK), possess excellent mechanical properties and biocompatibility; however, their inherently low surface energy limits effective bonding with resin cements. This study investigated the effects of hydrofluoric acid (HF) etching and handheld nonthermal plasma (HNP) treatment on enhancing the adhesive performance of PAEK surfaces. Disk-shaped PEEK (BP) and PEKK (PK) specimens were divided into four groups: APA (airborne-particle abrasion), PLA (nonthermal plasma treatment), LHF (5.0% HF), and HHF (9.5% HF). Surface characterization was performed using a thermal field emission scanning electron microscope (FE-SEM). Surface wettability was evaluated using contact angle goniometry. Cytotoxicity was evaluated using HGF-1 cells exposed to conditioned media and analyzed via PrestoBlue assays. Shear bond strength (SBS) was measured after three aging conditions—NT (no aging), TC (thermocycling), and HA (highly accelerated aging)—using a light-curing resin cement. Failure modes were categorized, and statistical analysis was performed using one-way and two-way ANOVA with Tukey’s HSD test (α = 0.05). Different surface treatments did not affect surface characterization. PLA treatment significantly improved surface wettability, resulting in the lowest contact angles among all groups, followed by HF etching (HHF > LHF), while APA showed the poorest hydrophilicity. Across all treatments, PK exhibited better wettability than BP. Cytotoxicity results confirmed that all surface treatments were nontoxic to HGF-1 cells, indicating favorable biocompatibility. SBS testing demonstrated that PLA-treated specimens achieved the highest and most stable bond strength across all aging conditions. Although HF-treated groups exhibited lower bond strength overall, BP samples treated with HF showed relatively less reduction following aging. Failure mode analysis revealed a shift from mixture and cohesive failures in the NT aging condition to predominantly adhesive failures after TC and HA aging conditions. Notably, the PLA-treated groups retained mixture failure patterns even after aging, suggesting improved interfacial durability. Among the tested methods, PLA treatment was the most effective strategy, enhancing surface wettability, bond strength, and aging resistance without compromising biocompatibility. In summary, the PLA demonstrated the greatest clinical potential for improving the adhesive performance of PAEK when used with light-curing resin cements.

## 1. Introduction

Polyaryletherketones (PAEKs) are a family of high-performance, semi-crystalline thermoplastic polymers characterized by excellent thermal stability and chemical resistance [1,2,3]. Their molecular structures are composed of numerous aromatic rings and ether and ketone linkages, forming a resonance-stabilized backbone with delocalized electrons [4,5]. This unique electronic configuration contributes to their superior heat resistance, mechanical strength, and chemical inertness, making PAEKs widely applicable in the aerospace, automotive, and biomedical fields [1,5,6]. Among the PAEK family, the most representative members are polyetheretherketone (PEEK) and polyetherketoneketone (PEKK), both of which exhibit high strength, chemical resistance, and favorable biocompatibility, although they differ slightly in molecular configuration [6,7]. While PEKK may demonstrate superior bonding performance in certain applications, its higher surface polarity and energy can limit its clinical utility [6,8]. In contrast, PEEK has gained widespread adoption in dentistry owing to its excellent toughness, elastic modulus comparable to dentin, and established processability, thereby becoming the most extensively used PAEK material in prosthetic and implant restorative applications [4,6,7,9,10].

PEEK exhibits an elastic modulus of approximately 4–8 GPa, closely matching that of human bone and dentin, allowing it to effectively distribute occlusal forces and reduce stress concentration at the interface between restorations and biological tissues, thereby demonstrating favorable biomechanical compatibility [2,9,11]. Additionally, its lightweight nature, high moldability, and resistance to corrosion have contributed to its increasing use in both fixed and removable dental prostheses [1,2,10]. In removable partial dentures, PEEK serves as a framework material offering a balance of aesthetics, comfort, mechanical stability, and low risk of metal hypersensitivity [10,12]. However, the intrinsic gray-white color of PEEK is not compatible with the natural tooth shade, limiting its use in highly aesthetic anterior restorations [2,6]. Clinically, composite resin veneers or ceramic-based restorations are often applied over PEEK surfaces to enhance aesthetic outcomes [3]. In the field of orthodontics, PEEK has also emerged as a potential alternative to stainless steel in the fabrication of lingual retainers and brackets, offering improved biocompatibility and aesthetic appeal [13,14].

Despite its many advantages, PEEK’s inherently low surface energy and chemical inertness hinder the formation of strong, durable bonds with resin-based materials [3,6,8,9]. Achieving stable adhesion is critical for the long-term clinical success of PEEK [15,16]. To address this challenge, ceramic-filled modified materials such as BioHPP (bredent medical GmbH & Co. KG) have been developed to enhance mechanical strength and confer some degree of bioactivity [1,4,9]. Nevertheless, even modified PEEK (BioHPP) struggles to establish robust chemical bonds with resin cements, necessitating the use of additional surface treatments to improve interfacial reactivity and bond strength [17,18,19,20]. Common surface modification strategies include airborne-particle abrasion [21,22,23], laser ablation [24,25], and etching with concentrated sulfuric acid (98% H_2_SO_4_) [25,26,27], all of which increase surface roughness and surface energy to promote mechanical interlocking and enhance the bonding interface. Among these, sulfuric acid etching effectively alters surface topography and introduces reactive functional groups, but its high corrosivity and associated safety concerns limit its clinical applicability [25,28]. Laser ablation, while efficient, may induce surface carbonization or irreversible structural damage, potentially compromising bonding performance [29].

The pursuit of a surface treatment strategy that balances safety and effectiveness forms the basis of this study. The current study investigates two promising surface treatment strategies—hydrofluoric acid (HF) etching, which presents a lower hazard level, and handheld nonthermal plasma (HNP) treatment—to evaluate their effects on the surface characteristics and resin bonding performance of polyetheretherketone. HF reacts with silica (SiO_2_) in silicate ceramics to produce highly reactive hexafluorosilicic acid, which induces micro-etching on the material surface, increasing surface roughness and wettability [30,31]. This reaction also promotes the exposure of silanol (–Si–OH) groups, creating active bonding sites that strengthen the chemical interactions between PAEK and resin adhesives, thereby improving overall bond durability [30,31]. Plasma treatment, a non-destructive physical modification method, generates high-energy reactive species from gas excitation and introduces polar functional groups such as carboxyl (–COOH), hydroxyl (–OH), and amide (–CONH_2_) groups [8,32]. Our previous studies have demonstrated that HNP significantly enhances the surface energy and wettability of PEEK, resulting in improved PEEK–resin adhesion [8,32].

In this study, two clinically relevant HF concentrations (5.0% and 9.5%) and HNP treatment are applied to modify the surface of PAEK specimens, and the results are compared with those obtained from conventional airborne-particle abrasion. Shear bond strength (SBS) tests are performed using light-curing cement, and artificial aging protocols are employed to simulate intraoral conditions and assess the long-term durability of the bonding. Ultimately, this study aims to establish a clinically feasible surface treatment protocol for PAEK-based lingual retainers or brackets, enhancing their adhesion to cement and thus improving their clinical applicability and performance.

## 2. Materials and Methods

### 2.1. Sample Preparation and Surface Treatment

Disk-shaped specimens (10 mm in diameter, 2.5 mm in thickness) were designed using 3D modeling software (SOLIDWORKS 2013, Dassault Systèmes SolidWorks Corp., Waltham, MA, USA) and fabricated from PEEK (breCAM BioHPP, bredent medical GmbH & Co. KG, Senden, Germany; BP) and PEKK (Pekkton ivory, Cendres+Métaux SA, Biel, Switzerland; PK) using a dental 5-axis milling unit (Milling Unit M1, Zirkonzahn GmbH, Gais, Italy). All specimens were polished with #600 silicon carbide paper to ensure uniform surfaces, ultrasonically cleaned in isopropanol, and dried in a 37 °C oven under sterile conditions. The specimens were then randomly divided into 4 groups (n = 50 per group) and subjected to different surface treatments, as listed in Table 1.

### 2.2. Surface Characterization

Surface characterization was performed using a thermal field emission scanning electron microscope (FE-SEM; JSM-7800F Prime, JEOL Ltd., Tokyo, Japan). Prior to observation, each sample was sputter-coated with a platinum layer. Imaging was conducted under high-vacuum conditions (9.6 × 10⁻^5^ Pa) with an accelerating voltage of 3.0 kV in secondary electron mode. Microstructural details were recorded via the instrument’s lower secondary electron detector.

### 2.3. Wettability

The surface wettability of specimens was evaluated using a contact angle analyzer (Phoenix Mini, Surface Electro Optics Co., Ltd., Gyeonggi-do, Republic of Korea) based on the sessile drop method. A 50 µL droplet of distilled water was gently placed in contact with the specimen surface at room temperature, allowing it to spread naturally. The droplet profile was captured in real time using a charge-coupled device (CCD) camera, and contact angles were calculated using a drop-shape fitting algorithm. Each specimen was measured three times, and a total of ten specimens per group (n = 10) were analyzed. The reported contact angle values represent the mean of ten independent measurements.

### 2.4. Cytotoxicity Testing

All specimens (n = 5) were sterilized by autoclaving at 121 °C and 1.2 kg/cm^2^ for 30 min, then dried under sterile conditions. Human gingival fibroblast-1 (HGF-1) cells (ATCC CRL-2014) were seeded into culture wells at a density of 1 × 10^5^ cells/well and cultured in high-glucose Dulbecco’s Modified Eagle Medium (DMEM) supplemented with 10% fetal bovine serum, 100 μg/mL streptomycin, and 100 U/mL penicillin. Cultures were maintained at 37 °C in a humidified atmosphere with 5% CO_2_, and the medium was refreshed every three days. Cells at passages 3–7 and full confluence were used for experiments. For cytotoxicity evaluation, specimens were immersed in DMEM at 37 °C for 72 h. HGF-1 cells were seeded into culture wells, and the culture medium was replaced with the conditioned DMEM collected from the specimen immersion. After 48 h of incubation, cell viability was assessed using the PrestoBlue™ cell viability reagent (Invitrogen, Carlsbad, CA, USA) following the manufacturer’s instructions. All experiments were performed in triplicate to ensure reproducibility. Optical density (OD) values were normalized against the control group (DMEM only), and the relative growth rate (RGR) was calculated using the formula RGR (%) = OD_sample_/OD_DMEM_ × 100.

### 2.5. Bonding Procedure and Shear Bond Strength (SBS) Testing

Before bonding, all specimens were primed with HC primer (SHOFU Inc., Kyoto, Japan) and light-cured for 90 s using a UV curing unit (Phrozen Tech Co., Ltd., Hsinchu City, Taiwan). A bonding ring was positioned on each specimen, and light-curing cement (Transbond XT, 3M ESPE Dental Products, St. Paul, MN, USA) was dispensed into it. A 4.9 N load was applied from above to ensure uniform cement thickness, and excess cement was removed prior to final light-curing using an LED curing unit. Specimens were then stored in a 37 °C oven to complete polymerization. The bonded specimens were divided into three aging groups (n = 10 each):

(1) NT—stored in distilled water at 37 °C for 24 h;
(2) TC—subjected to 5000 thermocycles (5–55 °C) [33];
(3) HA—subjected to highly accelerated aging (134 °C, 2 bar, 5 h) [34].

SBS testing was conducted using a universal testing machine (JSV-H1000, ALGOL Instrument Co., Ltd., Taoyuan, Taiwan) by applying a shear force at the adhesive interface at a crosshead speed of 1 mm/min until failure. Shear bond strength was calculated for all specimens. Failure modes were examined under a dental magnifier and classified as adhesive, cohesive, or mixture failure.

### 2.6. Statistical Analyses

The minimum sample size was calculated using power analysis software (GPower v3.1.9.6; Heinrich Heine University, Düsseldorf, Germany) based on previously reported surface characterization, cytotoxicity, and SBS data [8,22,32,35]. Data are expressed as mean ± standard deviation. Normality was assessed using the Shapiro–Wilk test, and parametric tests were applied. One-way analysis of variance (ANOVA) followed by Tukey’s honestly significant difference (HSD) post hoc test was used to analyze contact angle, surface energy, and cytotoxicity. SBS values across different surface treatments and aging conditions were analyzed using two-way ANOVA followed by Tukey’s HSD test. Statistical analyses were performed using SPSS (v19; IBM Corp., Armonk, NY, USA) and GraphPad Prism (v10; GraphPad Software Inc., San Diego, CA, USA), with the significance level set at α = 0.05.

## 3. Results

### 3.1. Surface Characterization

**Figure 1:**
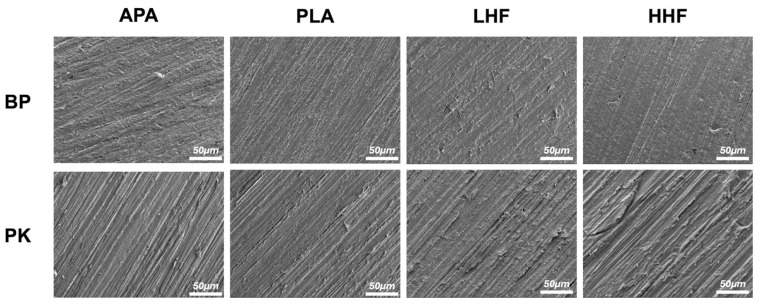
The FE-SEM surface morphologies of PEEK (BP) and PEKK (PK) specimens after different surface treatments. APA: airborne-particle abrasion; PLA: nonthermal plasma treatment; LHF: low-concentration hydrofluoric acid etching; HHF: high-concentration hydrofluoric acid etching.

The microscopic surface morphologies are presented in Figure 1, revealing no distinct changes across different treatments. APA produced grooves and uneven marks on both BP and PK, with PK showing deeper and more defined patterns. PLA led to moderate roughening with visible linear features, while LHF and HHF caused minor surface irregularities on BP but induced more pronounced erosion and peeling on PK, especially under HHF.

### 3.2. Wettability

**Figure 2:**
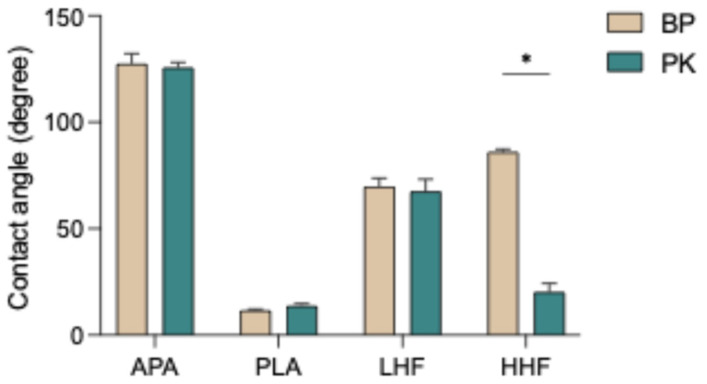
Water contact angles of PEEK (BP) and PEKK (PK) specimens after different surface treatments. APA: airborne-particle abrasion; PLA: nonthermal plasma treatment; LHF: low-concentration hydrofluoric acid etching; HHF: high-concentration hydrofluoric acid etching. The asterisk symbol (*) indicates that there is a significant difference (*p* < 0.05) between the two materials (BP and PK) under the same surface treatment conditions.

The results of surface wettability, as shown in Figure 2 and Table 2, indicate that the APA group exhibited the highest contact angles, indicating the poorest wettability. In contrast, the PLA group significantly reduced the contact angle, suggesting a substantial improvement in surface hydrophilicity. HF etching also effectively enhanced wettability, with both the LHF and HHF groups showing lower contact angles than the APA group. Notably, high-concentration HF etching (HHF) resulted in greater hydrophilicity compared with low-concentration HF (LHF). Regarding material differences, PK consistently exhibited lower contact angles than BP under all treatment conditions, indicating higher surface responsiveness and superior wettability. Overall, plasma treatment was the most effective at enhancing surface wettability, followed by HF etching, while airborne-particle abrasion was the least effective.

### 3.3. Cytotoxicity Test

**Figure 3:**
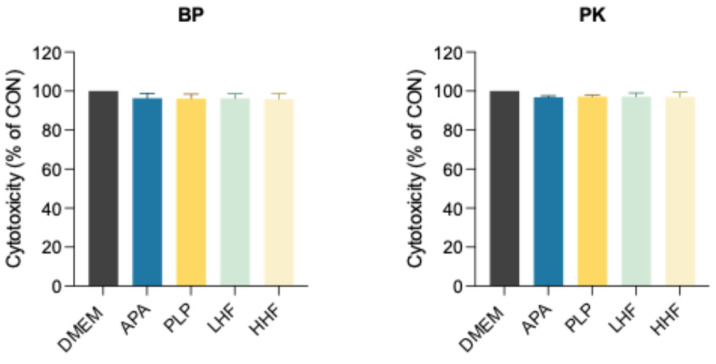
Assessment of cytotoxicity activity by indirect exposure of HGF-1 cells to extracts, consisting of conditioned DMEM collected from the immersion of PEEK (BP) and PEKK (PK) specimens treated with different methods using the PrestoBlue cell viability assay.

The cytotoxicity results (Figure 3) showed that both BP and PK specimens exhibited comparable cell viability across all surface treatments, with no significant differences between groups (*p* > 0.05), and the results were comparable to the untreated control (DMEM only), indicating no detectable cytotoxic effects.

### 3.4. SBS Testing

**Figure 4:**
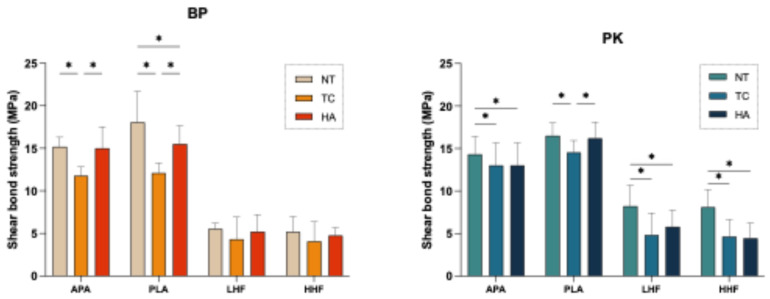
Effects of different surface treatments and aging conditions on the shear bond strength (SBS) of PEEK (BP) and PEKK (PK). NT: stored in distilled water at 37 °C for 24 h; TC: subjected to 5000 thermocycles between 5 °C and 55 °C; HA: subjected to highly accelerated aging at 134 °C and 2 bar for 5 h. The asterisk symbol (*) indicates that there is a significant difference (*p* < 0.05) between the aging conditions (NT, TC, and HA) under the same surface treatment conditions.

Figure 4 and Table 2 illustrate the changes in SBS across different surface treatment groups under three artificial aging conditions. Following the TC aging condition, all groups exhibited a significant decrease in SBS (*p* < 0.05), except for the HF-treated groups (LHF and HHF) in the BP. Despite the general reduction, the PLA group maintained a relative advantage (*p* < 0.05). Under the HA aging condition, the decline in SBS was less pronounced than under the TC aging condition, with BP exhibiting a more substantial reduction compared with PK across all surface treatments. Nevertheless, the PLA group consistently exhibited superior performance, with the highest and most stable SBS values across all aging conditions. In contrast, the HF-treated groups (LHF and HHF) showed the lowest SBS values both before and after aging. However, it is noteworthy that the HF-treated BP specimens exhibited only slight decreases in SBS following aging. Furthermore, PK generally exhibited higher SBS values than BP under all conditions.

### 3.5. Debonded Interface Analysis

**Figure 5:**
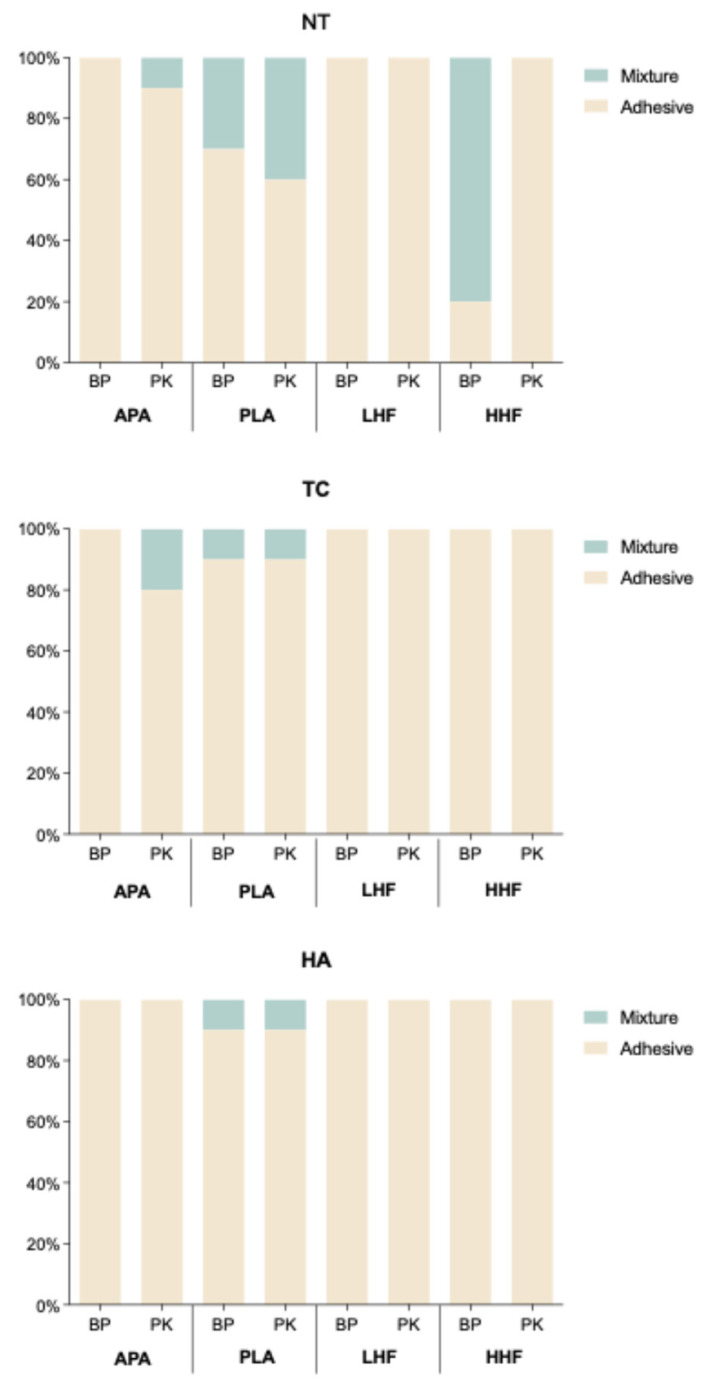
Distribution of failure modes (adhesive, mixture, and cohesive) for PEEK (BP) and PEKK (PK) under various surface treatments and aging conditions (NT, TC, and HA). NT: stored in distilled water at 37 °C for 24 h; TC: subjected to 5000 thermocycles between 5 °C and 55 °C (ISO10477); HA: subjected to highly accelerated aging at 134 °C and 2 bar for 5 h (ISO13356).

Debonded interface analysis (Figure 5) revealed that, in the NT condition, mixture and cohesive failures were observed in the BP-PLA and BP-HHF groups as well as in the PK-APA and PK-PLA groups. In contrast, adhesive failure predominated in the LHF groups. Following the TC aging condition, the proportion of adhesive failures increased. Nonetheless, mixture failures were still present in the PK-APA group and in the PLA-treated groups of both materials. Under the HA aging condition, most specimens exhibited adhesive failure, although the PLA-treated groups of both materials retained some instances of mixture failure.

## 4. Discussion

This study evaluated the effects of different surface treatments (Table 1)—airborne-particle abrasion (APA), nonthermal plasma treatment (PLA), and low-concentration (LHF) and high-concentration HF etching (HHF)—on the surface wettability, cytotoxicity, SBS, and bonding durability after artificial aging of PEEK (BP) and PEKK (PK). The results demonstrated that PLA exhibited the best overall performance across all parameters, while PEKK consistently outperformed PEEK, providing important implications for clinical applications.

Although SEM imaging (Figure 1) revealed some surface topographical differences across treatments, the overall morphological variations were relatively subtle, particularly for BP. APA and PLA induced mild surface roughening, while LHF and HHF caused limited morphological changes. The less-pronounced differences suggest that bonding performance may be more influenced by chemical modifications than by surface roughness alone, especially in chemically inert substrates like BP.

The surface wettability results revealed that PLA effectively reduced the water contact angle (Figure 2), significantly enhancing the hydrophilicity of the material surface. This finding aligns with previous studies reporting that plasma treatment introduces hydrophilic functional groups (e.g., hydroxyl, carboxyl) onto polymer surfaces, thereby increasing surface energy [8,17,21,32]. In contrast, APA increased surface roughness only through physical mechanisms without altering the surface chemistry, resulting in higher contact angles (Figure 2 and Table 2) and limited improvement in wettability [22,36]. HF etching also decreased contact angles, with high-concentration etching yielding greater hydrophilicity than low-concentration etching, likely due to the stronger etching effect of high-concentration HF, which exposes more reactive sites on the surface [30]. The modification mechanism of HF etching on PEEK may involve partial fluorination or hydrolysis reactions with the ketone (–C=O) and ether (–O–) groups in the polymer backbone, leading to the formation of hydroxyl (–OH), carboxyl (–COOH), or fluorocarbon (C–F) bonds on the surface, thereby increasing surface polarity and wettability [25,28,30]. In addition, HF etching may induce localized micro-etching, increasing the surface roughness and forming micropores or irregular structures that contribute to mechanical interlocking with resin. However, due to the aromatic structure and high crystallinity of BP, its chemical reactivity is relatively limited, resulting in a restricted increase in surface functional groups after HF etching [30,31]. Across all treatment conditions, PK exhibited lower contact angles than BP (Table 2), indicating superior surface reactivity, which may be attributed to its higher ketone content that enhances sensitivity to surface modification. In addition, the current study found that HHF etching significantly reduced the contact angle on PK surfaces, while an opposite trend was observed for BP containing nano-ceramic fillers (Figure 2). This difference may be attributed to variations in polymer structure and filler composition. PK, with a higher ketone content and rigid backbone, undergoes more pronounced surface erosion under HF treatment, resulting in increased roughness and enhanced hydrophilicity. In contrast, the ceramic fillers in BP are less reactive to HF and may lead to uneven surface degradation, filler exposure, or interfacial instability during etching, ultimately reducing wettability. These findings suggest that the interaction between the polymer matrix and fillers plays a critical role in surface modification outcomes.

Regarding biocompatibility (Figure 3), this study found that all treatment groups exhibited cell viability comparable to the untreated control, with no significant cytotoxic effects, indicating that neither the physical nor chemical treatments left harmful residues on the materials, and their potential impact on soft tissues is relatively safe. This finding corroborates previous reports on the biocompatibility of surface-modified BP and PK, supporting the clinical applicability of these treatment methods [11,28,32].

In terms of bonding performance, PLA achieved the highest initial SBS and maintained stable and superior SBS across all aging conditions (Figure 4), indicating its efficacy in enhancing interfacial bonding strength and durability (Table 2). After the TC aging condition, all groups showed significant decreases in SBS (*p* < 0.05), except for the HF-treated BP groups (LHF, HHF). Nevertheless, the PLA-treated groups retained a relative advantage (*p* < 0.05), demonstrating resistance to thermal cycling stress at the bonding interface. Under the TC aging condition (Figure 4), the reduction in SBS was even greater than under the HA aging condition, with BP showing more pronounced declines; however, PLA still maintained a relative advantage, confirming its superior aging stability. In contrast, the HF-treated groups exhibited the lowest SBS both before and after aging, suggesting limited improvement in bonding performance (Figure 4). Notably, the SBS reduction in the HF-treated groups after aging was not significant, indicating that their aging resistance remained relatively unchanged. This result may be attributed to the introduction of C–F, –OH, and –COOH functional groups by HF etching on the BP surface, which increased wettability but did not effectively form chemical crosslinks with resin monomers (e.g., MMA, UDMA). Moreover, excessive etching may have weakened the mechanical integrity and introduced interfacial microcracks, thereby limiting the overall bonding performance improvement [19,30,31].

The failure mode analysis (Figure 5) supported the SBS findings. In the NT aging condition, mixture and cohesive failures were observed in the BP-PLA and BP-HHF groups as well as in the PK-APA and PK-PLA groups, indicating sufficient interfacial bonding strength to propagate failure into the resin or substrate. In contrast, adhesive failure predominated in the APA and LHF groups. After the TC aging condition (Figure 5), the proportion of adhesive failures increased overall, suggesting thermal stress weakened the bonding interface; however, the PLA-treated groups still exhibited some mixture failures, reflecting higher resistance to aging. Under the HA aging condition (Figure 4), most specimens exhibited adhesive failure, but the PLA-treated groups of both materials retained some mixture failures, corresponding to the superior SBS results and further supporting the correlation between failure mode and bonding durability.

In summary, PLA demonstrated superior performance in improving the surface wettability, resin bonding, and post-aging bonding stability of both BP and PK while maintaining safety and efficacy, thereby offering a clinically viable surface treatment strategy. Although HF etching increased surface polarity and wettability and introduced microstructural roughness, its contribution to stable chemical bonding was limited, and the potential adverse effects of over-etching on mechanical integrity must be considered. Particularly in BP, despite the detection of C–F, –OH, and –COOH groups after HF treatment, the inert polymer backbone and aromatic ring stability hindered the achievement of functional group density and reactivity comparable to plasma treatment. Furthermore, PK consistently outperformed BP under all treatment and aging conditions, indicating that its higher ketone content and structural characteristics contribute to more stable chemical bonding with resin, making it a more promising candidate for clinical applications requiring long-term bonding stability. While this study independently assessed plasma treatment and HF etching, it did not evaluate their combined application. Future research could explore whether sequential plasma activation followed by HF etching yields synergistic benefits, combining enhanced chemical reactivity and modified surface morphology to further improve interfacial bonding and durability between polymer substrates and resin. This approach may offer a promising surface treatment strategy for high-performance polymers.

## 5. Conclusions

This study confirmed that surface treatments significantly influenced the surface characteristics, biocompatibility, and resin bonding performance of BP and PK. All treatments were non-cytotoxic. Among them, PLA showed the best overall results, enhancing wettability and achieving the highest and most stable SBS across all aging conditions. While HHF improved wettability, its bond durability was limited. PK outperformed BP in wettability, bonding strength, and aging resistance. Notably, only PLA-treated groups retained mixture failures after aging, indicating better interfacial stability. Overall, PLA is the most effective and clinically feasible surface treatment for improving the adhesive performance of PAEK materials.

## Figures and Tables

**Table 1:** Surface treatment protocols for each experimental group.

Group	Surface Treatment Methods
APA	Airborne-particle abrasion with 110 µm alumina (Cobra, Renfert GmbH, Hilzingen, Germany) at 2 bars for 10 s.
PLA	Handheld nonthermal plasma treatment (PiezoBrush PZ3, Relyon plasma GmbH, Regensburg, Germany) for 10 s.
LHF	5.0% hydrofluoric acid etching (IPS Ceramic Etching Gel, Ivoclar Vivadent, Schaan, Liechtenstein) for 120 s.
HHF	9.5% hydrofluoric acid etching (Porcelain Etchant, Bisco Inc., Schaumburg, IL, USA) for 120 s.

**Table 2:** Results of water contact angles and SBS.

Material	Surface Treatment	Contact Angle (Degrees)		Shear Bond Strength (MPa)		
				NT	TC	HA
BP (PEEK)	APA	127.41 ± 4.72	^a^	15.18 ± 1.10 ^a^	11.76 ± 1.04 ^a^	15.01 ± 2.53 ^a^
	PLA	11.66 ± 0.52	^b^	18.06 ± 3.63 ^b^	12.09 ± 1.18 ^b^	15.51 ± 2.15 ^a^
	LHF	69.76 ± 3.88	^c^	5.51 ± 0.66 ^c^	4.31 ± 2.60 ^c^	5.21 ± 1.99 ^b^
	HHF	86.06 ± 1.26	^d^	5.17 ± 1.79 ^c^	4.07 ± 2.30 ^c^	4.75 ± 0.91 ^b^
PK (PEKK)	APA	125.66 ± 2.37	^A^	14.31 ± 2.10 ^A^	13.01 ± 2.65 ^A^	13.01 ± 2.65 ^A^
	PLA	13.79 ± 0.98	^B^	16.51 ± 1.51 ^B^	14.53 ± 1.41 ^B^	16.19 ± 1.94 ^B^
	LHF	67.55 ± 5.68	^C^	8.22 ± 2.45 ^C^	4.81 ± 2.61 ^C^	5.80 ± 1.95 ^C^
	HHF	20.30 ± 3.98	^D^	8.08 ± 2.06 ^C^	4.64 ± 1.99 ^C^	4.44 ± 1.79 ^C^

All the results are presented as average ± standard deviation. Artificial aging condition: NT, stored in distilled water at 37 °C for 24 h; TC, subjected to 5000 thermocycles between 5 °C and 55 °C; HA, subjected to highly accelerated aging at 134 °C and 2 bar for 5 h. Different letters (a, b, c, d for BP and A, B, C, D for PK) in the same column indicate significant differences (*p* < 0.05).

## Data Availability

The original contributions presented in this study are included in the article. Further inquiries can be directed to the corresponding authors.
